# Application of thermogravimetric analysis (TGA) and differential scanning calorimetry (DSC) to estimate the normal boiling points of ∆9-tetrahydrocannabivarin (THCV) and ∆9-tetrahydrocannabinol (THC)

**DOI:** 10.1186/s42238-025-00373-w

**Published:** 2025-12-09

**Authors:** Eli H. Turovsky, Kyle Moriarty, Nicole Chavarria, James E. Parco, Remy Kachadourian, Murphy G. Brasuel

**Affiliations:** 1 Department of Chemistry and Biochemistry, Colorado College, Colorado Springs, CO USA; 2 Environmental Studies and Science Department, Colorado College, Colorado Springs, CO USA; 3 Schwazze Biosciences, Denver, CO USA

**Keywords:** ∆9-tetrahydrocannabivarin, Δ9-tetrahydrocannabinol, Normal boiling point, Heats of vaporization, Thermogravimetric analysis, Differential scanning calorimetry

## Abstract

**Background:**

Physical properties, particularly boiling point and heat of vaporization are important when designing cannabinoid purification strategies based on distillation methods and when engineering vaporization-based cannabinoid delivery commercial products.

**Methods:**

Cannabinoid standards were obtained from Sigma-Aldrich. Concentrates of ∆9-tetrahydrocannabivarin (THCV), Δ9-tetrahydrocannabinol (THC), and THCV-THC mixtures were obtained from Schwazze Biosciences. Thermogravimetric Analysis (TGA) and Differential Scanning Calorimetry (DSC) were used to determine boiling points and estimate heats of vaporization (*ΔH*_vap_).

**Results:**

Based on combined TGA and DSC data and the Sydney-Young equation, the 95% confidence interval for THCV normal boiling point was 378 ± 4 °C. The 95% confidence interval for THC normal boiling point was 245 ± 6 °C.

**Discussion:**

Experimental values for boiling point were significantly different from the values commonly reported for both compounds in commonly referenced sources. The values in popular literature and websites are often not referenced and do not indicate pressures for reported boiling points.

## Introduction

Minor non-psychotropic phytocannabinoids have demonstrated potential therapeutic efficacies. The purification and biological delivery of these phytocannabinoids is aided by knowledge of both their physical and chemical properties. Of particular interest in this work is ∆^9^-tetrahydrocannabivarin (THCV). THCV is a minor cannabinoid with low psychotropic effect. THCV has been shown to act as an antagonist of cannabinoid receptor 1 (CB1) in vivo and in vitro and a partial agonist of cannabinoid receptor 2 (CB2) at low concentrations (Pertwee 2008). THCV can activate Transient Receptor Vanilloid 1 (TRPV1), and Transient Receptor Vanilloid 2 (TRPV2) (De Petrocellis et al. 2011). THCV acts as agonist of G-protein coupled receptor-55 (GPR55) (Wargent et al. 2020). THCV has been shown to interact with the mitogen-activated protein kinase signaling pathway (MAPK) leading to the reduction of pro-inflammatory cytokines (Tortolani et al. 2023). Interactions with these receptors makes THCV a good candidate for efficacy in the treatment of obesity and diabetes (Pertwee 2008; Tudge et al. 2014; Jadoon et al. 2016; Abioye et al. 2020; Wargent et al. 2020), treatment of insulin insensitivity due to obesity (Wargent et al. 2013), treatment of skin inflammation (Tortolani et al. 2023), treatment of dyskinesia in Parkinson’s disease (García et al. 2011; Espadas et al. 2020), and treatment of seizures (Hill et al. 2010).

THCV is commonly found in a mixture with the psychotropic cannabinoid Δ^9^-tetrahydrocannabinol (THC), therefore the formulation of pure THCV products requires separation methods to isolate THCV from THC. A key physical characteristic is the boiling point of the pure cannabinoids. Knowing the boiling point of the pure cannabinoids is extremely useful when optimizing parameters for distillation purification methods and calculating expected boiling points of mixtures. It is common for chemical supply companies to provide normal (1 atm or 1.013 bar) physical properties in the accompanying safety data sheets (SDS). The SDS sheets supplied with the THC standard from Millipore/Sigma lists the normal boiling point as having no data available (Millipore/Sigma 2024). A standard Internet search yields numerous websites purporting the boiling point of THC to be 157 °C (314.6 °F) and the boiling point of THCV to be 220 °C (428 °F). This information is presented with scant reference and often fails to establish the pressure conditions under which these boiling points are purported to be true. Some websites claim the data comes from the Environmental Protection Agency (EPA) CompTox Chemicals Dashboard (https://comptox.epa.gov/dashboard/). Yet, the dashboard for THC for physchem properties reports the predicted average normal boiling point of THC as 375 °C and the predicted range of probable THC boiling point from 328 °C to 407 °C, based on four different computational methods for predicting normal boiling point based on chemical structure. It can be assumed that the 157 °C boiling point for THC reported on many websites likely references a boiling point at reduced pressure and not a boiling point at atmospheric pressure. A survey of the literature revealed that the early work on the isolation of natural and synthetically modified/produced cannabinoids reported 157 °C as the appropriate temperature to isolate THC by distillation at a pressure of 0.05 Torr (Adams et al. 1941; Gaoni and Mechoulam 1964). There currently are no physicochemical properties listed for THCV in the EPA CompTox Chemicals Dashboard. The purported 220 °C boiling point of THCV likely references the distillation temperature at 0.05 Torr as it is often listed in comparison to a 157^°^C boiling point for THC but no references detailing the experimental measurement conditions of this value were found in the literature.

Determining the true boiling points of THCV and THC by thermal analysis methods not only addresses the lack of well-referenced data in scientific literature, but it also can guide differential distillation. In 1994, Jones and Seyler established Differential Scanning Calorimetry (DSC) as a rapid, accurate method to determine boiling points using milligram quantities of sample (Jones and Seyler 1994). Goodrum further established that DSC and Thermogravimetric Analysis (TGA) can provide similar boiling point results using similar sample preparation methods (Goodrum and Siesel 1996). Goodrum demonstrated the measurement of boiling points by TGA and DSC for short-chain triglycerides and saturated medium- and long-chain triglycerides (Goodrum 1997; Goodrum and Geller 2002) thus supporting the use TGA and DSC methods to determine the atmospheric pressure boiling points of THC and THCV.

### Purification methods

*Extraction and decarboxylation of cannabinoids*: Extraction of cannabinoids was carried out from dried and ground floral plant material (high THCV strain, 3:1 - THCVA: THCA, PTC8505V from Phylos Bioscience, Portland, OR) using a Cup-30 cold ethanol extractor (Delta Separations, Cotati, CA). The extraction solvent was 200 proof food grade ethanol (Rocky Mountain Reagents, Golden, CO). The ethanol was evaporated using a Falling Film Evaporator (Delta Separations) and the resulting extract was treated at 110 °C under vacuum for 8 h for the decarboxylation of cannabinoids and elimination of residual ethanol using a 50 L glass reactor under vacuum system from Keda Instruments (Zhengzhou, China). Raw floral plant on average was 15.3% THCVA (Tetrahydrocannabivarinic Acid) and 9.0% THCA (Tetrahydrocannabinolic acid). Average extraction yield was 12.7% by mass with average extract composition having 42.8% THCV and 34.0% THC.

*Distillation of cannabinoids*: Cannabinoid distillate was obtained using a 6-inch stainless-steel wiped-film evaporator system from Pope Scientific (Saukville, Wisconsin). Working parameters for distillation were 120–165 °C heating mantle temperature (HMT), 80–90 °C internal condenser temperature (ICT) at a pressure of 180 mTorr.

*Purification of cannabinoids*: THCV was purified using a Selekt Biotage flash chromatography system equipped with a Biotage Sfär C18 400 g column (CV, dead volume, = 582 mL) and a UV detector (230 nm) and using an ethanol and water (65:35) mix as mobile phase.

### Analytical methods

Heptadecane 99% pure, liquid at room temperature (CAS# 629-73-7), ethyl linoleate ≥ 99%, liquid at room temperature (CAS# 544-35-4), cannabinoid mixture standard (Cerilliant^®^, 1 mg/mL in methanol each of (−)-trans-Δ9-THC, cannabidiol, and cannabinol in methanol) product number C-220 and GC grade methanol (CAS# 67-56-1) were obtained from Sigma-Aldrich. THCV and THC were obtained from Schwazze Biosciences. Three cannabinoid mixtures, mixture 1 (*85.75% cannabinoid*,* 16.75% THC*,* 0.00% CBD*,* 0.91% CBG*,* 0.53% CBC*,* 0.00% CBN and 67.46%*), mixture 2 (*90.49% cannabinoid*,* 44.16% THC*,* 0.00% CBD*,* 1.90% CBG*,* 0.55% CBC*,* 0.00% CBN and 43.91% THCV*) and mixture 3 (*89.79% cannabinoid*,* 23.72% THC*,* 0.00% CBD*,* 1.01% CBG*,* 0.75% CBC*,* 0.00% CBN and 64.31% THCV*) were also obtained from Schwazze Biosciences. Cannabinoids and cannabinoid mixtures were a viscous liquid at room temperature.

Extraction, distillation and purification of cannabinoids performed at Schwazze Biosciences were monitored with high pressure liquid chromatography (HPLC). THCV and THC levels in cannabis extract and distillate were monitored using an Agilent 1100 HPLC system equipped with a Phenomenex Kinetex 2.6 mm C18 100 A (150 × 4.6 mm) column (Torrance, CA) and an isocratic mobile phase of a mix acetonitrile/water with 0.1% phosphoric acid (75:25), and a flow of 1.0 ml/min (12 min total run); the retention times of THCV and THC were 4.2 and 7.0 min, respectively.

Confirmation of THC and THCV cannabinoid purity at Colorado College was performed by gas chromatography-mass spectroscopy (GC-MS) analysis. Samples were prepared for GC-MS analysis by dissolving cannabinoids in GC-MS grade methanol. Cannabinoid standard and samples were analyzed on an Agilent 7890 A GC system coupled to an Agilent 5975 C inert XL MSD with an Agilent 19,091 S-433, HP-5MS, 5% phenyl methyl silox. 30 m x 250 μm x 0.25 μm column. A 3 µL injection with a 50:1 split was utilized. The Injector temperature was set to 250 °C. The column temperature program started at 40 °C with a one-minute hold and then a ramp to 220 °C at 30 °C/min, ramp to 260 °C at 10 °C/min, hold for 5 min, and a final ramp to 300 °C at 30 °C/min.

Thermogravimetric analysis (TGA) of phytochemical (heptadecane and ethyl linoleate) and cannabinoid boiling points were performed on a Waters TA Discovery TGA 5500 instrument and Differential Scanning Calorimetry (DSC) analysis of phytochemical and cannabinoid boiling points and heats of vaporization (*ΔH*_vap_) were performed on a Waters TA Discovery DSC 250 instrument. Between three and fifteen mg of sample, mixed with 2–3 mg of alumina diluent (when used) were prepared in Tzero pans with an internal volume of 20 µL (TA instruments part number 901683.901). Heptadecane and ethyl linoleate are non-viscous liquids at room temperature and were transferred to Tzero pans with a small volume pipette. For viscous cannabinoid samples, the samples were loaded into small volume glass syringes. These syringes were heated to 50 °C in an oven and sample was transferred from the heated syringe into Tzero pans warmed on top of a hot plate to 50 °C. The heating allowed for clean transfer of sample. The Tzero pans were hermetically sealed with Tzero hermetic pinhole lids with a 75 μm diameter laser drilled pinhole (TA instruments part number 901685.901). The instrument (TGA or DSC) temperature ramp was set between 5 °C/min and 10 °C/min for each analysis.

For TGA experiments boiling point was determined by using the instrument software to find the onset temperature of the mass lost feature on TGA graph, see Fig. 2A. For DSC experiments the onset temperature of the enthalpy of vaporization peak gives the boiling point, see Fig. 2B (Goodrum and Siesel 1996) The heat of vaporization (*ΔH*_vap_) is determined from the total energy in joules in the integration of the vaporization peak divided by the total mass of the original sample to give *ΔH*_vap_ in J/g. This number is corrected for the mass of sample lost in reaching the boiling point as determined from TGA experiments run using the same parameters as the DSC experiments (Rojas-Aguilar et al. 2005) and then converted to *ΔH*_vap_ in units of KJ/mol. On average heptadecane experienced 25% mass loss, ethyl linoleate experienced 26% mass loss, THCV experienced 25% mass loss, and THC experienced 22% mass loss in reaching their respective boiling points.

### Data method verification

The two phytochemicals heptadecane and ethyl linoleate were selected as test compounds for boiling point determination of pure compounds and mixtures by TGA and DSC analysis. Very-long-chain (VLC) alkanes and long-chain (LC) alkanes are commonly produced by plants through biosynthetic pathways (Wen et al. 2025). Heptadecane (C_17_H_36_) is one such LC-alkane and is a common phytochemical found in plant extracts. Heptadecane has a normal boiling point between 288.50 and 301.85 °C (Brown and Stein 2025) with a *ΔH*_vap_ = 86.5 KJ/mol (Acree Jr. and Chickos 2025). Ethyl linoleate (C_20_H_36_O_2_) is a common phytochemical known as an essential fatty acid. It is found in high concentrations in garlic and is one of phytochemicals that gives garlic anti-inflammatory properties (Park et al. 2014). Ethyl linoleate has an estimated normal boiling point of 355.6 °C (Wallek et al. 2018) with a *ΔH*_vap_ = 72.6 KJ/mol (Acree Jr. and Chickos 2025).

The atmospheric pressure during TGA and DSC analysis of heptadecane and ethyl linoleate ranged between 610.0 and 615.0 mm Hg. See Fig. 2 for representative TGA and DSC data. The average atmospheric pressure was recorded during each run using a mercury barometer. Within a reasonable range of atmospheric pressures, the heat of vaporization is constant. Normal boiling points (at 1 atm) were calculated by two methods for comparison. The first using data at barometric pressure recorded during the time of the experiment, the average experimentally determined *ΔH*_vap_ and the Clausius-Clapeyron equation (see Eq. 1).

1 $$\:\:Ln{p}_{2}=Ln{p}_{1}+\left(\frac{{\varDelta\:H}_{v}}{R}\right)\left(\frac{1}{{T}_{1}}-\frac{1}{{T}_{2}}\right)$$

Where p_1_ and p_2_ are vapor pressure (atm) at temperatures T_1_ and T_2_ (Kelvin). *ΔH*_vap_ is the heat of vaporization (KJ/mole) and R is the gas constant (0.00831447 KJ K^-1^  mol^-1^). Boiling occurs when vapor pressure equals atmospheric pressure.

The second method used data at barometric pressure recorded during the time of the experiment and the Sydney-Young equation (Young 2010).

2 $$\:\:{\Theta\:}=0.00012\times\:(760-p)\times\:(273+t)\:$$

Θ is the correction in centigrade or kelvin to add to the observed boiling point to obtain the boiling point at 760 mm Hg, p is the pressure in mm Hg of the observed boiling point and t is the observed boiling point. See Table 1 for summarized data using both analysis methods.

**Table 1:** Normal boiling points as determined by TGA and DSC for heptadecane and ethyl linoleate ^a^ calculated from Clausius-Clapeyron equation ^b^ calculated from Sydney-Young equation. Expected heptadecane boiling point and *ΔH*_vap_ are 301.85 °C and 86.5 KJ/mol respectively. Expected ethyl linoleate boiling point and *ΔH*_vap_ are 355.6 °C and 72.6 KJ/mol respectively

Compound	∆H_vap_ (kJ/mol) DSC	bp °C TGA	bp °C DSC	Expected bp °C
Heptadecane	47._9_±22._3_ (± 95%CI, *n* = 3)	307._6_±2._3_^a^ 305._4_±2._3_^b^ (± 95%CI, *n* = 3)	305.0_0_±0.8_5_^a^ 302.8_3_±0.8_3_^b^ (± 95%CI, *n* = 3)	288.50–301.85 (Brown and Stein 2025)
Ethyl linoleate	30._1_±11._2_ (± 95%CI, *n* = 3)	374.4_8_±0.7_3_^a^ 361.7_9_±0.6_9_^b^ (± 95%CI, *n* = 3)	371._5_±1._3_^a^ 358._7_±1._2_^b^ (± 95%CI, *n* = 3)	355.6 (Wallek et al. 2018)

The experimental determined boiling points of heptadecane and ethyl linoleate give a reasonable estimate of the expected boiling points based on literature values with best data coming from use of the Sydney-Young equation. For both test compounds the experimentally measured heats of vaporization are lower than expected by 55% for heptadecane and by 41% for ethyl linoleate.

If it can be assumed that when two volatile compounds form an ideal solution, then Raoult’s law (see Eq. 2) can be used to determine the vapor pressure of two component mixture).

3 $${p}_{T}=\:{{p}_{a}X}_{a}={{p}_{b}X}_{b}$$

The boiling point of the mixture occurs when p_T_ equals atmospheric pressure. Equations 1 and 3 are utilized in a system of equations to predict the boiling point of a two-component mixture. The less ideal the solution the greater the deviation between predicted and measured boiling point for the mixture. The boiling point of three mixtures of heptadecane and ethyl linoleate were prepared were measured by TGA (see Table 2). The predicted boiling points as determined using Eqs. 1 and 2 with DSC data from Table 1 were compared to the measured boiling point. This system of equations accurately predicted the boiling points of the mixtures within ± 2.5%.

**Table 2:** Predicted and measured boiling points of heptadecane and ethyl linoleate mixtures at 610.1–614.2.1.2 mmhg

	Mixture 1	Mixture 2	Mixture 3
Mole Ratio heptadecane: ethyl linoleate	60:40	50:50	30:70
Average bp (°C) (± SD, *n* = 3)	308._4_±1._0_	313._2_±1._3_	320._7_±3._6_
Predicted bp (°C)	302.5	306.0	315.2
%Error (Δ/Predicted)100	2.0%	2.4%	1.7%

The agreement between experimental TGA mixture boiling point measurements using normal boiling point values of the pure compounds determined by TGA and DSC experiments confirms these methods for determining the boiling points of cannabinoids. The validation experiments also indicate that these methods will require further optimization in order to accurately determine heats of vaporization. The heat of vaporization calculated by these experiments are systematically about 50% lower than expected. Likely the amount of material lost to vaporization before reaching the boiling point has been underestimated. Each different mass weighed into the Tzero pans will have a different surface area to volume ratio. Experimental data will be required for each substance to appropriately factor in how the surface area to volume ratio impacts the specific amount of material lost while the DSC instrument is heating up to the boiling point. Control experiments are also needed to determine the appropriate correction factor needed to account for the aluminum mass difference between the sample Tzero pan and the reference Tzero pan. Given the results of the method verification experiments the THCV and THC boiling points are determined using the Sydney-Young equation.

## Results and discussion

### Data analysis and discussion of the boiling point of THC and THCV

**Fig. 1:**
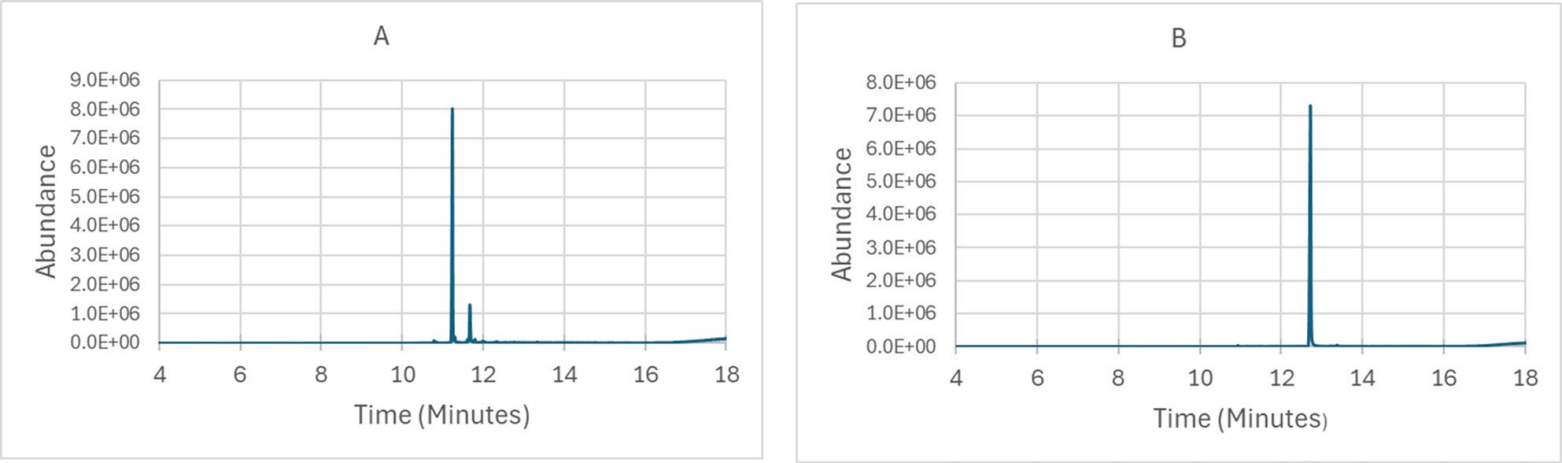
**A** GC-MS TIC for THCV concentrate, results indicate THCV concentrate was 84% THCV (RT:11.24 min) and 16% CBV (RT:11.67 min). **B** GC-MS TIC for THC concentrate, results indicate THC concentrate was 98% THC (RT:12.73 min)

THCV concentrate obtained from Schwazze Biosciences was 84% THCV and 16% CBV. The THC concentrate was 98% THC, see figure 1.

**Fig. 2:**
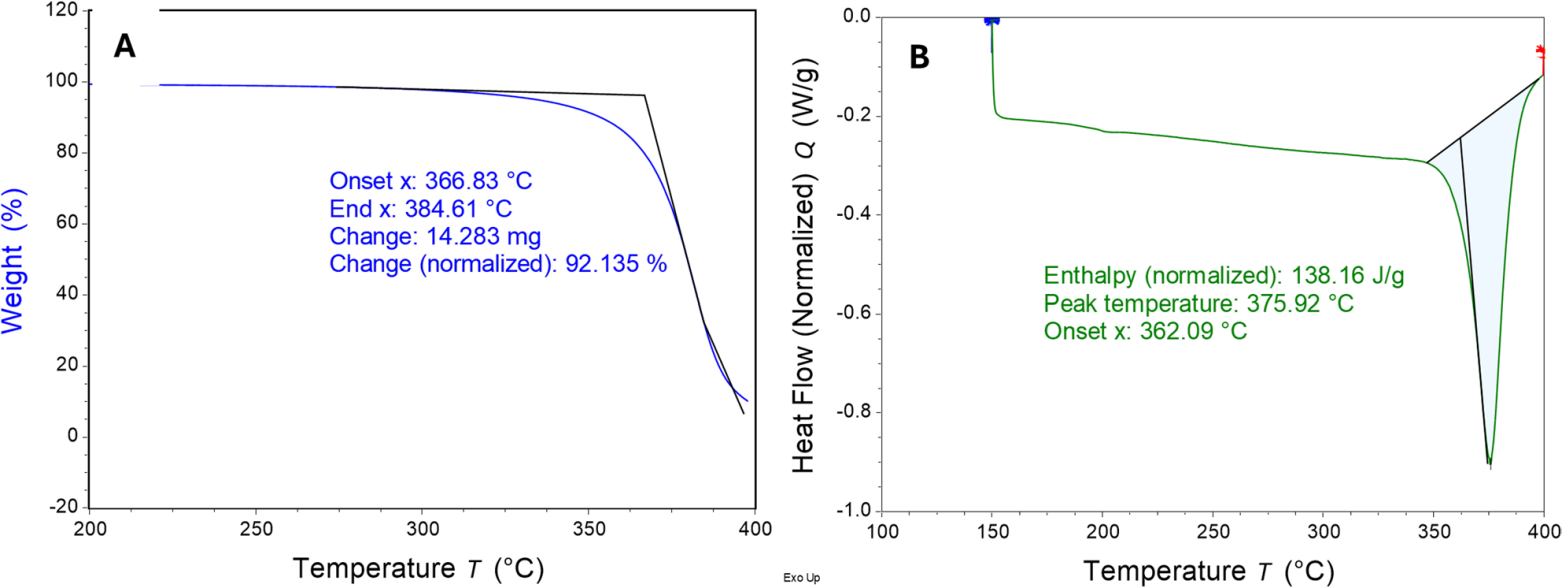
**A** TGA data for a THCV trial. Onset temperature for transition indicates boiling point. **B** DSC data for a THCV trial. Onset temperature for transition indicates boiling point. Integration of peak represents *ΔH*_vap_ in J/g for cannabinoid material left when sample reaches this temperature. We have no explanation for the artifact noted at about 200 °C

Atmospheric pressure during TGA and DSC analysis of THC and THCV ranged between 610.0 and 620.0 mm Hg. See Fig. 2 for representative TGA and DSC data for THCV.

Average atmospheric pressure was recorded during each run using a mercury barometer. Method verification indicated that heats of vaporization found using this methodology are significantly lower than expected. Thus, normal boiling points (at 1 atm) were calculated using (1) boiling point data and the barometric pressure recorded during the time of the experiment; (2) and the Sydney-Young equation (Eq. 2).

Based on the combined TGA and DSC data, the 95% confidence interval for THCV boiling point a 378 ± 4 °C and the 95% confidence interval for THC boiling point was 245 ± 6 °C. A system of equations combining Eqs. 1 and 2 was able to predict the boiling point of three cannabinoid concentrate mixtures of THC: THCV ranging from 44 to 67% THCV within ± 9.5% for a given barometric pressure (*n* = 8) despite heats of vaporization being low estimates (Table 3).

**Table 3:** Summary of the normal boiling points as determined by TGA and DSC calculated from Sydney-Young equation

Compound	bp ^o^C TGA	bp ^o^C DSC	bp ^o^C (purported)
THCV	376._8_±15._4_ (95%CI, *n* = 2)	377._9_±5._7_ (95%CI, *n* = 5)	220
THC	239._7_±1._6_ (95%CI, *n* = 3)	250._0_±9._0_ (95%CI, *n* = 3)	157

## Conclusion

The normal boiling points of THC and THCV are significantly different than the commonly purported boiling points of THC (157 °C) and THCV (220 °C). TGA and DSC measurements indicate boiling point values of 245 °C for THC and 378 °C for THCV at 760 mm Hg (1 atm) atmospheric pressure.

## Data Availability

The raw data obtained and analyzed during the current study are available from the corresponding author on reasonable request.

## Abbreviations

THCV: Δ9-tetrahydrocannabivarin
THC: Δ9-tetrahydrocannabinol
TGA: Thermogravimetric Analysis
DSC: Differential Scanning Calorimetry
GC-MS: Gas Chromatography-Mass Spectroscopy
